# Molecular Analysis of RNA-RNA Interactions between 5′ and 3′ Untranslated Regions during the Initiation of Translation of a Cardiovirulent and a Live-Attenuated Coxsackievirus B3 Strains

**DOI:** 10.3390/ijms14034525

**Published:** 2013-02-25

**Authors:** Amira Souii, Jawhar Gharbi, Manel Ben M’hadheb-Gharbi

**Affiliations:** 1 Laboratoire des Maladies Transmissibles et Substances Biologiquement Actives (LR99-ES27), Faculté de Pharmacie de Monastir, Avenue Avicenne, Monastir 5000, Tunisia; E-Mails: jawhargharbi@yahoo.fr (J.G.); benmhadhebmanel@yahoo.fr (M.B.M.-G.); 2 Institut Supérieur de Biotechnologie de Monastir, Université de Monastir, Avenue Tahar Hadded, BP 74, Monastir 5000, Tunisia

**Keywords:** CVB3, 5′UTR, 3′UTR, RNA-RNA interaction, electrophoretic mobility shift assay

## Abstract

Coxsackievirus B3 (CVB3) is a causative agent of viral myocarditis, meningitis and pancreatitis. CVB3 overcome their host cells by usurping the translation machinery to benefit viral gene expression. This is accomplished through alternative translation initiation in a cap independent manner at the viral internal ribosomal entry site. The 5′ untranslated region (5′UTR) of CVB3 genomic RNA is highly structured. It is the site of multiple RNA-protein and RNA-RNA interactions and it plays a critical role during translation initiation. Similar to the 5′UTR, CVB3 3′ untranslated region (3′UTR) also contains secondary structural elements consisting of three stem-loops followed by a poly (A) tail sequence. Long-range RNA-RNA interactions between 5′ and 3′ ends of some viral genomes have been observed. Because of their dual role in translation and replication, the 5′ and 3′UTRs represent promising candidates for the study of CVB3 cardiovirulence. Taking into account that efficient initiation of mRNA translation depends on a temporally and spatially orchestrated sequence of protein-protein, protein-RNA and RNA-RNA interactions, and that, at present, little is known about RNA-RNA interactions between CVB3 5′ and 3′UTRs, we aimed in the present study, to assess a possible RNA-RNA interaction between 5′ and 3′UTRs during the initiation of translation of a wild-type and a previously characterized mutant (*Sabin3-like*) CVB3 strains and to investigate the effect of the *Sabin3-like* mutation on these potential interactions. For this purpose, “Electrophoretic Mobility Shift” assays were carried out. Data obtained did not show any RNA-RNA direct interactions between the 5′- and 3′- ends. Therefore, we can suggest that the possible mechanism by which 3′UTR enhances CVB3 IRES activity may be by bridging the 5′ to the 3′ end through RNA-protein interaction and not through RNA-RNA direct contact. However, these findings need to be confirmed by carrying out further experiments.

## 1. Introduction

Coxsackievirus B3 (CVB3), a member of the family *Picornaviridae*, is the causative agent of virus-induced myocarditis and dilated cardiomyopathy [1,2]. The CVB3 RNA, like that of a typical picornavirus, contains a single, long open reading frame (ORF) flanked by 5′ and 3′ untranslated regions (UTRs) [3,4]. Unlike cellular messenger RNAs, translation of picornavirus genomes occurs via a cap-independent pathway. Initiation of protein synthesis in the eukaryotic cell leads to the assembly of the 80S ribosome at the start codon of the mRNA. At least two mechanisms for recruiting and positioning ribosomes on the mRNA have been described [5,6]. The primary mechanism involves the recognition of the 5′ cap structure (m7GpppN) by eukaryotic translation initiation factors (eIFs), followed by binding of the 40S ribosomal subunit and scanning downstream to the initiation codon [5–7]. Alternatively, in some mRNAs, a structural element named the “Internal Ribosome Entry Site” (IRES), allows assembly of the translational machinery at a position close to or directly at the initiation codon [8,9]. In fact, picornaviruses lack the 7-methyl G cap structure and instead have a viral protein (VPg) that is covalently linked to the 5′ terminus of the genome [10,11]. Additionally, during an enterovirus infection, the virus-encoded proteinase 2A cleaves eIF4G to disrupt the cap-binding complex [12,13]. Without a 5′ cap to initiate cap-recognition and ribosome scanning, picornaviruses utilize an alternative, internal pathway for translation initiation. The RNA secondary structures that form within the 5′ non coding region (NCR) serve as an internal ribosome entry site for translation initiation [14,15].

The exact mechanism of IRES-mediated translation initiation has not been clearly elucidated for the CVB3; however, it has been postulated that the interaction of trans-acting host factors with cis-acting stem-loop structures and helices acts to recruit canonical and non-canonical translation factors and/or stabilize the RNA for translation. The canonical factors that have been shown to play a role in IRES-mediated translation are eIF3, eIF4B and a cleaved part of eIF4G [9]. In addition, a number of non-canonical factors, known as IRES trans-acting factors or ITAFs, have been shown to have crucial influence on IRES-mediated translation. These include human La-autoantigen (p52), polypyrimidine tract-binding protein (PTB or p57) [16], upstream-of-NRas (Unr or p97) and poly (rC) binding protein-2 (PCBP2 or p39) [4,9,17,18].

The ~740-nucleotide CVB3 5′NCR is highly structured, containing multiple stem-loop elements [19,20]. It contains an IRES that directs internal initiation of translation of the coding region of the viral genome [19,21,22]. Ribosomes are recruited upstream of the AUG triplet at position 591 (AUG_591_), also called the cryptic AUG, after which they scan downstream for about 150 nt before initiating at the initiator AUG or AUG_741_[23–25]. Involvement of the local secondary structure in modulating IRES function has been reported in some viral RNAs [23,26–28].

**Figure 1:**
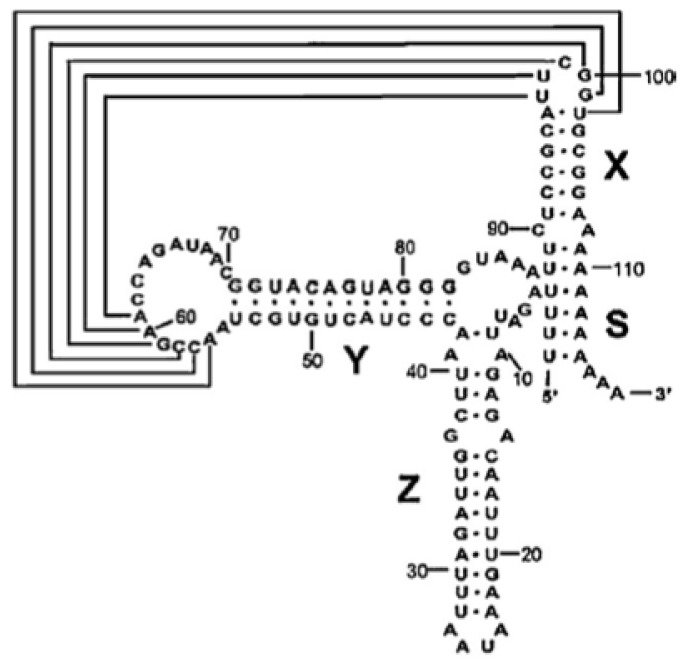
Structure of the Enterovirus 3′ untranslated region (3′UTR) and secondary structure of the individual domains X, Y, and Z [48].

Similar to the 5′UTR, the 3′UTR also contains secondary structural elements [29]. The 3′UTR of CVB3 is 99 nt long and highly structured, containing conserved domains, and is followed by a poly (A) tail of variable length [30]. The secondary structure of the 3′UTR of CVB3 RNA (Figure 1) consists of three stem-loops (X, Y and Z), followed by a poly (A) tail sequence. Interactions among these stem-loops enable the formation of kissing-pair tertiary structure facilitating viral translation and replication [31,32]. Nevertheless, the role of these stem-loops is not well known, but they are suggested to play an important role during regulation of virus replication through interaction with cellular proteins. The 3′UTR is known to enhance IRES activity, although the mechanism is not clear [4,31,33].

**Figure 2:**
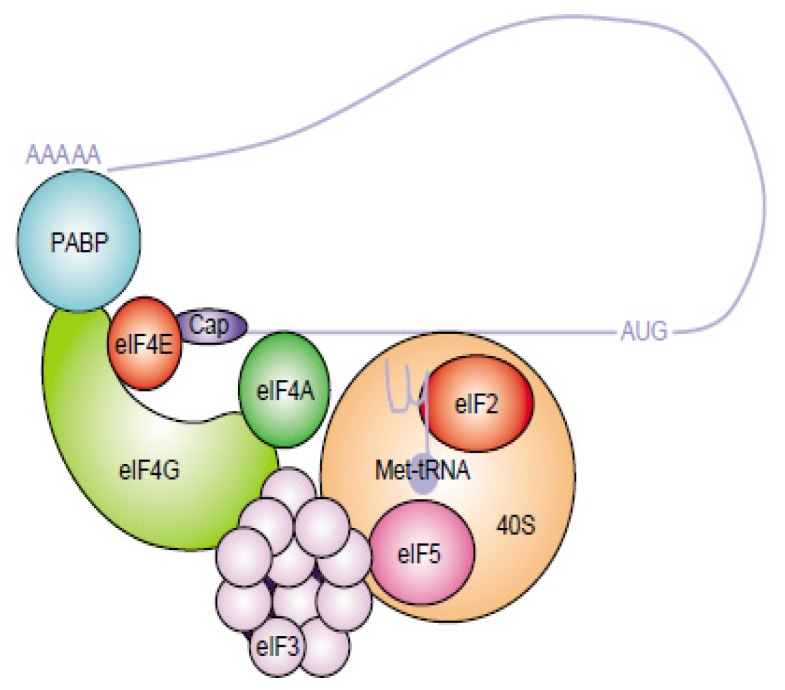
The Eukaryotic Translation Initiation Complex. Capped mRNAs are recruited by the eIF4F complex, which is composed of two subunits, eIF4E and eIF4G. eIF4E is the cap binding protein and binds the m^7^G cap structure at the 5′ end of mRNAs. eIF4G is a “scaffolding” protein and interacts with several other initiation factors, poly (A) binding protein (PABP), eIF4A (a DEAD box helicase for unwinding secondary structures) and eIF3 (a multisubunit complex that binds to the 40S ribosome). In the current model of initiation of protein synthesis of cellular mRNA, once the mRNA is bound to the 40S ribosome, the ribosome and associated factors [eIF2–Met–tRNA–GTP (ternary complex), eIF1, eIF1A] scan along the mRNA until an initiation codon in an acceptable context is encountered. eIF5-mediated GTP hydrolysis of the ternary complex occurs during the binding of the 60S ribosomal subunit to form the functional 80S ribosome. In the internal initiation model, direct interaction occurs between protein(s) of the initiation complex and mRNA without the need for an m7G cap structure [49].

Although the role of the CVB3 poly (A) tail is not very clear, knowledge from its cellular mRNA counterpart indicates that its presence may benefit RNA stability and enhance translation initiation [34,35]. In cellular mRNAs, the poly (A) tail can interact with the poly (A) binding protein (PABP) to mediate the formation of a closed loop for translation initiation (Figure 2). Whether this mechanism is employed by viral RNA needs to be investigated. Cheung and collaborators [36] reported that the poly (A) tail is a critical sequence mediating host protein interactions with CVB3 3′UTR through base-pairing with its preceding 3′UTR sequence. Together with the poly (A) tract, the 3′UTR has been implicated in both translation and replication [33,37,38]. Nevertheless, translation- and replication- competent picornaviruses have been generated with complete 3′UTR deletions [33,39]. These findings suggest that while specific 3′UTR sequences and/or secondary structures may promote or regulate translation and replication, they are not essential for viral viability [29].

Several strategies have been proposed for 5′-3′-end contacts in positive-strand RNA viruses, including rotaviruses, pestiviruses and picornaviruses, all involving RNA-binding proteins [40–45]. The possible mechanism by which the 3′UTR enhances IRES activity may be by bridging the 5′ to the 3′ end, through either RNA-RNA contacts or RNA-protein interaction. Interestingly, it has been shown for CVB3 that distal sequences, including 3′UTR and poly (A) tail sequences, stimulate IRES function [33]. It was also reported that CVB3 IRES-mediated translation was stimulated significantly by the 3′UTR *in cis*, both *in vitro* in rabbit reticulocyte lysate and *ex vivo* in HeLa cells [4]. Although the exact mechanism of this enhancement is not clear, it is possible that the rate of translation initiation mediated by the IRES element is increased by the 3′UTR.

In a previous study, Ben M’hadheb-Gharbi [46] reported the limited efficiency of translation of the CVB3 *Sabin3-like* strain. This mutant (U^473^→C), obtained by direct mutagenesis, had a significantly reduced translation capacity compared to the wild-type strain. Prediction of the secondary structure by MFOLD program indicated a structural perturbation of the stem containing the *Sabin3-like* mutation, suggesting that specific protein-viral RNA interactions were disrupted, preventing efficient viral translation. The poor translation efficiency of the *Sabin3-like* IRES was then explicated by a reduced affinity of the mutant RNA to correctly bind some essential non-canonical translation factors such as eIF3, p100 (the C-terminal two-thirds fragment of eIF4G which lacks an eIF4E binding site) and the 40S ribosomal subunit during the initiation of translation [47].

On the basis of the data that the CVB3 3′UTR stimulates the IRES translation initiation as above reported, it is tempting to speculate that one or more proteins may be involved in interacting simultaneously with the 5′ and 3′UTRs, thus bringing about circularization of the mRNA. Additionally, it is possible that some ITAF interaction, together with long range RNA-RNA interaction and RNA-protein interactions, might contribute to the efficient IRES activity of CVB3 RNA. Since there is no previous report on RNA-RNA interactions of the CVB3 and in order to assess the effect of the *Sabin3-like* mutation on these potential interactions, we examined, in the present study, the possibility of long-range RNA-RNA interactions between the 5′ and 3′ untranslated regions during the initiation of translation of the wild-type and the mutant CVB3 strains. For this purpose, 5′ and 3′ ends of CVB3 wild-type and *Sabin3-like* RNAs were labeled using the fluorescence and “Native Gel Shift” assays were carried out in order to investigate a possibility of RNA-RNA interactions between these two non coding regions.

## 2. Results

### 2.1. Cloning of the CVB3 5′ and 3′ Untranslated Regions

Two CVB3 strains were analyzed: a wild-type and an attenuated *Sabin3-like* strains. The attenuation of the *Sabin3-like* strain was mainly conferred by a single point mutation in the 5′UTR sequence and, precisely, in domain V of the IRES [46]. Wild-type and *Sabin3-like* 5′ UTRs were amplified and then cloned in a pUC19 vector between EcoRI/BamHI restriction sites as previously described in “Material and methods” section.

Additionally, in order to clone the CVB3 3′UTR, the complete 3′ non coding region within the poly (A) tail was synthesized using primers- hybridization and extension technique and then was cloned between EcoRI and HindIII restriction sites of the pUC19 plasmid. Transformed pUC19/5′UTRs and pUC19/3′UTR clones were confirmed by PCR-colony, then, cloned sequences and their orientations were verified by DNA sequencing.

### 2.2. *In Vitro* Transcription

The CVB3 3′- and 5′UTRs- pUC19 clones were linearized with EcoRI and used for *in vitro* transcription. Polyadenylated 3′UTR RNA was directly synthesized *in vitro* using T7 RNA polymerase while for the 5′UTR, DNA template was generated from the respective pUC19/5′UTR constructs by PCR using the T7 forward primer and a reverse primer containing the sequences corresponding to the AUG initiation codon and 15 nucleotides from the coding region. 5′UTR PCR-amplified products were used as template for the *in vitro* transcription reaction as described above. RNAs were then precipitated, purified using exclusion chromatography technique, quantified and labeled with fluorescence.

### 2.3. RNA and Fluorescent Labeling

To assess a possibility of RNA-RNA interactions between 5′ and 3′ non coding regions during the CVB3 translation initiation, the 5′UTR RNA was labeled with HiLyte Fluor™ 680 which is an excellent amine-reactive fluorescent labeling dye that generates protein conjugates that are only slightly red-shifted. Its fluorescence emission is well separated from that of other commonly used red fluorophores. For this purpose, the 3′ terminus of wild-type and mutant 5′UTR RNAs was oxidized by sodium periodate and then labeled with HiLyte Fluor™ 680.

**Figure 3:**
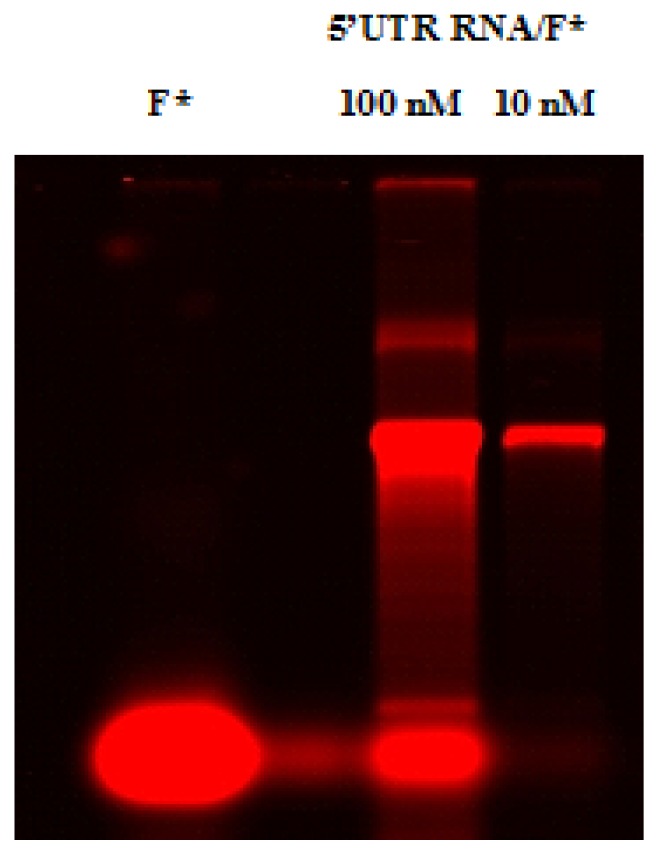
Fluorescent labeling of the Coxsackievirus B3 5′UTR RNA. The 5′UTR RNA labeled with HiLyte Fluor™ 680 (lane 5′UTR RNA/F*) was loaded in a syber-free 1.5% agarose gel and visualized with Scanner Odyssey (Li-COR Odyssey, Biosciences). For a further comparative analysis of electrophoretic pattern of the labeled 5′UTR RNA and for an optimization of the amount of labeled RNA needed for the gel shift assay, two RNA concentrations were loaded (10 and 100 nM). Taking into account the appropriate molar ratio RNA/Fluorophore and a minimum amount of free fluorophore (lane F*) that may reduce the efficiency of the gel shift reaction, we can deduce that 10 nM is the optimal concentration to carry out further gel shift experiments.

Following a fluorescent labeling reaction, it is often necessary to remove any non reacted fluorophore from the labeled target molecule. This is usually accomplished by size exclusion chromatography, taking advantage of the size difference between fluorophore and labeled RNA. Taking into account that fluorophores may interact with the separation matrix and reduce the efficiency of separation, specialized dye removal columns that account for hydrophobic properties of fluorescent dyes are sometimes used. G50 Sephadex columns purification were used for this purpose. Labeled RNAs were loaded in a syber-free 1.5% agarose gel (Figure 3), visualized with Scanner Odyssey and quantified using Biospec-NanoDrop technology.

### 2.4. Native Gel Shift Assay Analysis

The Electrophoretic Mobility Shift assay (EMSA) is a commonly used technique for studying *in vitro* protein-RNA or RNA-RNA interactions. It is based on the fact that RNA-protein or RNA-RNA complexes migrate slower in a non-denaturing gel than the free RNA. When a protein-RNA or RNA-RNA complex is formed, the migration of the complex is slower. Furthermore, the corresponding band migrates far less on the gel and is therefore known as “shifted”. This shift allows us to conclude that a particular RNA sequence has been recognized and bound by a protein or an RNA.

**Figure 4:**
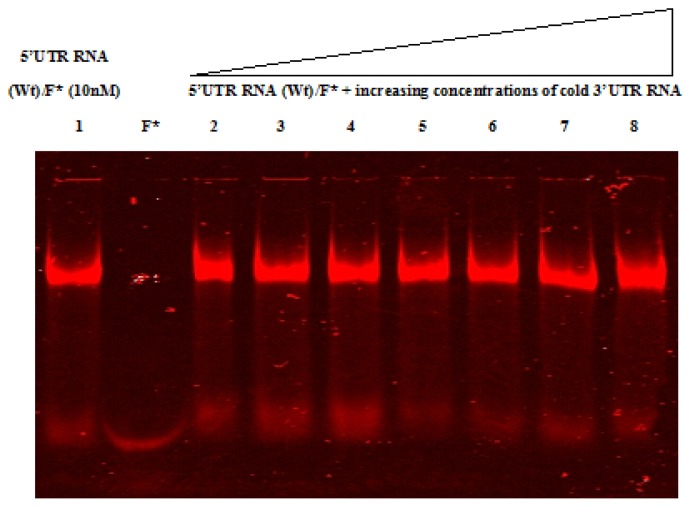
Electrophoretic Mobility Shift assay of the wild-type 5′UTR and the 3′UTR of Coxsackievirus B3. *In vitro* transcribed HiLyte Fluor™ 680-5′UTR wild-type RNA (5′UTR RNA (Wt)/F*) (lane 1) at a final concentration of 10 nM was incubated with increasing amounts of cold 3′UTR RNA (lanes 2–8). Reactions were then resolved by a native polyacrylamide gel (4%) electrophoresis. Lane F* represents the free fluorophore (HiLyte Fluor™ 680). According to this electrophoretic pattern visualized with Scanner Odyssey (Li-COR Odyssey, Biosciences), the 3′UTR RNA is unable to interact within the wild-type 5′UTR RNA.

**Figure 5:**
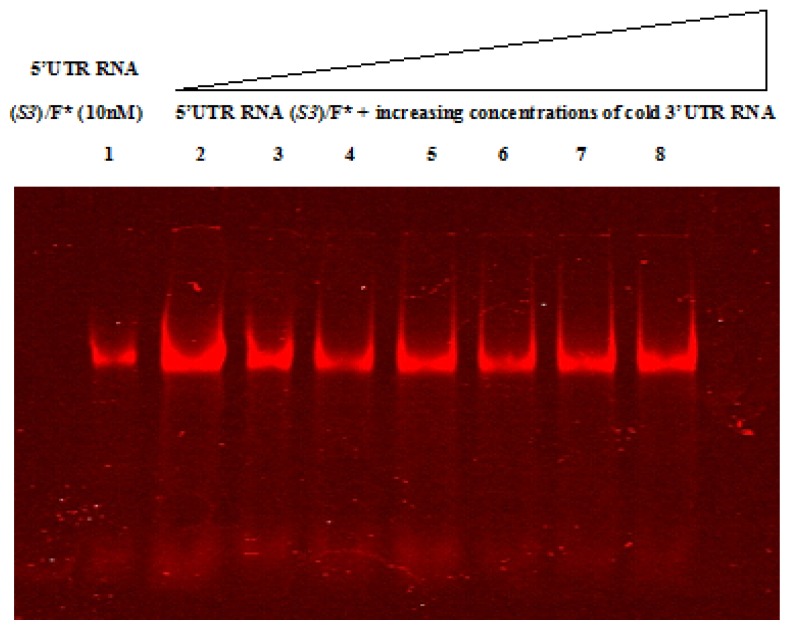
Electrophoretic Mobility Shift assay assessing RNA-RNA interactions between the *Sabin3-like* 5′UTR and the 3′UTR of Coxsackievirus B3. *In vitro* transcribed HiLyte Fluor™ 680-5′UTR *Sabin3-like* RNA (5′UTR RNA (S3)/F*) at a final concentration of 10 nM (lane 1) was incubated with increasing amounts of cold 3′UTR RNA (lanes 2–8). Reactions were then subjected to a native polyacrylamide gel (4%) electrophoresis and the electrophoretic pattern was visualized using the Scanner Odyssey (Li-COR Odyssey, Biosciences). As we can see, the 3′UTR RNA is unable to interact within the *Sabin3-like* 5′UTR RNA.

RNA-RNA electrophoretic mobility shift assays were performed using labeled CVB3 wild-type and mutant 5′UTRs and cold 3′UTR to assess a possibility of interaction between these two regions. RNAs were analyzed on 4% polyacrylamide native gel (Figures 4 and 5). Free labeled 5′UTR RNA was used as a negative control. The 5′ and 3′ non coding regions of Hepatitis C virus (HCV), a positive single-stranded RNA virus whose protein synthesis is also initiated in an IRES-dependent manner, were used as control probes (data not shown), since an RNA-RNA interaction between these two structures has been previously reported [50], in parallel to CVB3 RNAs. Figures 4 and 5 show that all RNA bands migrate at the same level in all lanes; suggesting the absence of a shift under the tested experimental conditions. The same result was obtained after incubation either at 4 °C or 37 °C. Taking into account this data, we can suggest that the 5′UTR did not directly interact within the 3′UTR. Nevertheless, further experiments should be performed in order to confirm this result.

## 3. Discussion

Immediately after uncoating in the cytoplasm, secondary structures that form the Coxsackievirus B3 5′UTR RNA recruit both canonical and non-canonical translation factors to mediate cap-independent translation. By hijacking cellular components for their own gene expression, picornaviruses exploit an alternative mechanism of internal initiation of translation that already exists in the cell. The interaction of IRES *trans*-acting factors (ITAFs) with RNA secondary structures has been shown to stimulate picornavirus IRES-mediated translation. Several RNA binding proteins have been identified as ITAFs, including PCBP2, the La autoantigen, *unr* (upstream of N-*Ras*), and polypyrimidine tract binding protein (PTB) [51]. The interaction of ITAFs with IRES elements may provide a functional equivalent to eIF4F binding to a 5′ cap structure, which picornaviruses lack, as well as an RNA chaperone like function to stabilize IRES structures [52]. The CVB3 genome encodes a single long open reading frame flanked by a 5′ and 3′ non coding regions. The IRES element in the 5′UTR RNA interacts with multiple host factors for translation initiation. The 3′UTR of the genomic RNA, containing extensive stretches of conserved residues, also contributes to IRES-mediated translation [4].

Because of their dual role in translation and replication, the 5′ and 3′UTRs represent promising candidates for the study of CVB3 cardiovirulence. RNA-RNA interactions between these two regions assist in these processes. Long-range RNA-RNA interactions between 5′ and 3′ ends of some viral genomes have been observed [45,50,53]. Consistent with a functional link between the ends of the viral RNA, IRES activity is stimulated by the 3′UTR [38,54].

Biochemical characterization of RNA-RNA interactions provides direct experimental evidence of tertiary interactions within the IRES element and constitutes the molecular basis to understand IRES-dependent translation initiation. In this report, we conducted a study aimed at understanding the involvement of the CVB3 3′-terminal region in the viral IRES stimulation and the effect of the *Sabin3-like* mutation on these possible RNA-RNA interactions. Since efficient initiation of mRNA translation depends on a temporally and spatially orchestrated sequence of protein-RNA and RNA-RNA interactions, and taking into account that, at present, little is known about RNA-RNA interactions between CVB3 5′ and 3′UTRs, we hypothesized the possibility of long-range RNA-RNA interactions between these two non coding regions. In this regard, electrophoretic mobility shift assays were carried out. RNA-RNA complexes were allowed to form at 4 °C and simultaneously at 37 °C to normalize the efficiency of the interactions under the study.

According to the obtained gel pattern, we did not observe any shift under the tested conditions, and consequently, no direct interactions between 5′ and 3′UTRs were noticed for both wild-type and *Sabin3-like* CVB3 RNAs. However, complementary assays should be carried out in order to validate this finding, especially that formation of RNA-RNA complexes is highly dependent on RNA concentration, ionic strength, and temperature, suggesting a dynamism in RNA interactions that may be related to the IRES efficiency. Interestingly, our result should not be surprising since Martìnez-Salas and collaborators [53] have reported recently that the bridging of 5′ and 3′ ends involves direct RNA-RNA contacts or RNA-protein interactions. Thus, it provides a mechanistic basis for translation stimulation and replication of the viral RNA resembling the synergistic stimulation of cap-dependent translation. Therefore, we can suggest that the possible mechanism by which the 3′UTR enhances CVB3 IRES activity may be by bridging the 5′ to the 3′ end, through RNA-protein interaction and not through RNA-RNA direct interaction. Nevertheless, the potential for RNA-RNA interactions between these two regions in the presence of specific proteins still exists (*i.e.*, in the context of a whole cell lysate and complete viral genome). In fact, RNA-protein interactions are required to organize the IRES element in the appropriate structure recognized by the translational machinery; while long-range tertiary interactions are essential for the proper folding and function of large biologically active RNAs. To date, the requirements of tertiary structure for recognition of the CVB3 IRES element by the trans-acting factors are still unknown. Thus, it is possible that these interactions are transient, being displaced by RNA binding proteins.

In the same context, in order to get better insights on the effect of *Sabin3-like* mutation on cellular protein interaction within the IRES and to identify proteins interacting with CVB3 wild-type and mutant RNAs, we have previously carried out UV cross-linking assays using labeled RNAs in the presence of either HeLa or BHK-21 *S10* extracts [47]. We have observed a number of proteins that specifically interact with both RNAs. In particular, molecular weights of five of these proteins resemble to those of the eukaryotic translation initiation factors 4G, 3b, 4B and PTB. Moreover, we have demonstrated a better affinity of CVB3 RNA binding to BHK-21 proteins and a reduced interaction of the mutant RNA with almost cellular polypeptides compared to the wild-type IRES.

On the basis of phylogeny of some initiation factors and on the knowledge of the initiation of translation process, we focused on the interaction of both IRESes with eIF3, p100 (eIF4G) and 40S ribosomal subunit by “Filter Binding assays”. We have demonstrated a better affinity of binding to the wild-type CVB3 IRES [47]. These results implicate that the main effect of the CVB3 *Sabin3-like* mutation in the viral IRES that contributes to the attenuation of CVB3 cardiovirulence and the reduction of translation efficiency is the impaired binding of standard translation initiation factors. This may cause slower translation of the viral RNA in some tissues and thus may contribute to the attenuated phenotype of the CVB3 mutant strain. Thus, the reduction efficiency of the mutant RNA to bind to cellular proteins involved in the translation initiation could be the reason behind inefficient IRES function. Hence, the identification of RNA-protein and RNA-RNA interacting network, is clearly a challenge in the near future to increase our understanding of how these specialized RNA structures perform their function.

Our present findings for RNA-RNA interactions between the CVB3 5′ and 3′UTRs are in correlation with data obtained by Cheung and collaborators [36] who reported that several proteins, particularly the La autoantigen, interact with 3′ and 5′UTRs mediating a cross-talk between these two non coding regions of CVB3 RNA. In the same context, as observed with La autoantigen, Verma and collaborators [4] showed the importance of a versatile cellular protein, PTB, in CVB3 IRES-mediated translation. They demonstrated that the protein interacts with conserved residues on both 5′ and 3′UTRs of the genomic RNA, possibly leading to circularization of the genomic RNA, and thereby influences the efficiency of IRES-mediated translation.

PTB showed multiple points of interaction on the 5′UTR as well as the 3′UTR of CVB3. A few interaction points on the 5′UTR were observed in the pyrimidine-rich region, while others were observed in AU-rich regions. Interestingly, a significant number of interaction points were found around the cryptic AUG (AUG_591_) on stem-loop H (SL-H) of the CVB3 IRES. Similar PTB contact points, rich in AU nucleotides, were also observed on the 3′UTR at regions that are highly conserved among different strains [37]. This suggests that the interaction between the 3′UTR and PTB might be a conserved feature among different Coxsackievirus strains.

Although PTB was observed to interact with both 5′ and 3′UTRs, the affinity of interaction with the former was observed to be higher than that with the latter [4]. This could be because of the higher number of interaction points in the 5′UTR, although the implications of such a different affinity are not yet clear. It would be interesting to investigate which domains of the PTB protein are responsible for binding to the different UTRs of CVB3 RNA, and whether PTB is able to bind both of them simultaneously. It would be interesting to study whether PTB and La binding influence ribosome loading on the CVB3 IRES RNA.

Moreover, The PTB interaction points on the CVB3 3′UTR were observed to be predominantly in Z and X domains (shown in Figure 1). Interestingly, partial deletion of either domain led to a significant drop in translation enhancement by the 3′UTR [4]. It may be possible that the interaction of PTB protein with the 3′UTR RNA is responsible for the effect of the 3′UTR on translation. Further, we can hypothesize that a simultaneous interaction of PTB protein with the 5′UTR and the 3′UTR might lead to circularization of the template RNA, leading to a higher rate of initiation. Consequently, the role of these proteins in 3′UTR-mediated translation enhancement cannot be ruled out and needs to be improved.

It was previously reported that the FMDV 3′UTR stimulates IRES activity, even in the absence of a poly (A) tail [54], suggesting a functional link between the IRES, which resides at the 5′end, and the 3′ end of the genomic viral RNA. It was shown that regions located at the 5′ and 3′ ends of the FMDV genome interact through strand-specific RNA-RNA contacts [45]. Two different elements, S and IRES, present at the 5′ end, interacted with the 3′UTR in a dose-dependent manner. No interactions were observed with the 3′UTR of swine vesicular disease virus (SVDV), a picornavirus that produces similar symptoms in infected animals. A high-order RNA structure adopted by both the complete IRES and the 3′UTR was essential for RNA interaction, whereas the S region could interact with each of the 3′UTR stem-loops. Additionally, they detected specific cellular proteins interacting with the S region. One of these, p47, competed for binding to the 3′UTR. These data on FMDV 5′–3′-end bridging, involving both direct RNA-RNA contacts and RNA-protein interactions, provide a mechanistic basis for the stimulation IRES activity mediated by specific viral RNA 3′-end sequences. The possibility that proteins might further stabilize RNA-RNA bridges in the FMDV genome is open.

Genome circularization promoted essentially by a single RNA-RNA interaction has been shown in Flaviviruses [55]. In this case, inverted terminal repeats had been reported [56,57]. It has been also shown that several proteins, including elongation factor 1-α, La and PTB, interact with Flavivirus 3′UTR [58]. Direct RNA-RNA interaction between a small number of residues in 3′ and 5′UTR sequences has been demonstrated to control cap-independent translation initiation in RNA plant viruses [59–61] that may be reminiscent of 5′–3′-end interaction in cap-dependent translation initiation of cellular mRNAs [62]. Similarly, long-range RNA-RNA interactions were also reported for the HCV [50,63].

Although previous studies in animal models have succeeded in identifying genetic modifications that attenuate the cardiovirulence of Coxsackieviruses B3, attempts to understand the underlying mechanism of virulence attenuation have not been undertaken. This is because the initiation of translation of these viruses involves a series of complex overlapping processes. CVB3 genome has evolved to accomplish multiple and often simultaneous functions in viral translation. Interestingly, the dual involvement of 5′ and 3′UTRs in controlling viral RNA translation and virus replication highlights the relevance of these regions in the infectious virus life cycle, making them suitable candidates for targeted antiviral therapy.

## 4. Experimental Section

### 4.1. Virus

The Coxsackievirus B3 (CVB3) Nancy prototype strain and the *Sabin3-like* mutant of CVB3, used as a “vaccine candidate” and obtained by direct mutagenesis [46] were used for all experiments. These strains were propaged in Vero cells (African Green Monkey Kidney Cells) (Bio Whittaker) maintained in Eagle’s Minimal Essential Medium (MEM) supplemented with 10% heat-inactivated Fetal Calf Serum (FCS) (Sigma), 1% L-Glutamine, 50 μg/mL de Streptomycin, 50 UI/mL de Penicillin (Bio Whittaker), 1% nonessential amino acids (Gibco BRL) and 0.05% Fongizone (Amphotericin B, Apothecon).

### 4.2. Plasmids

The pUC19 (plasmid of University of California) (Invitrogen) was used for cloning 5′ and 3′UTRs of both CVB3 strains. It is one of the most widely used vector molecules as recombinants and can be easily distinguished from the non-recombinants based on colour differences of colonies on growth media. It has one *ampR* gene (Ampicillin Resistance gene), and an N-terminal fragment of β-galactosidase (*lacZ*) gene of *Escherchia coli*. It carries a 54 base-pair Multiple Cloning Site (MCS) polylinker that contains unique sites for 13 different hexanucleotide-specific restriction endonucleases. The *lacZ* fragment, whose synthesis can be induced by IPTG (Isopropyl βeta-d-ThioGalactopyranoside). In the presence of IPTG in growth medium, bacteria synthesize both fragments of the enzyme that can together hydrolyze the X-gal (5-Bromo-4-Chloro-3-Indolyl- βeta-D-Galactopyranoside) and form blue colonies on media with X-gal. Thus, bacteria carrying recombinant plasmids in the MCS cannot hydrolyse the X-gal, giving rise to white colonies.

### 4.3. Bacterial Strains

*Escherichia (E.) coli* DH5α strain was transformed with recombinant plasmids, and was cultured in Luria Bertani (LB) broth to select transformants. This strain has many mutations that make it useful for transformation. Its genotype is [*dlacZ DeltaM15 Delta (lacZYA-argF) U169 recA1 endA1 hsdR17 (rK-mK+) supE44 thi-1 gyrA96 relA1*] but the most useful of these mutations is the *lacZ Delta M15* mutation which allows blue/white screening for recombinant cells.

### 4.4. Viral RNA Extraction

Total RNA was extracted using the Acid Guanidinium Thiocyanate Phenol-Chloroform method [64]. After ethanol precipitation, the total RNA was resuspended in DEPC (DiEthyl PyroCarbonate) treated sterile water.

### 4.5. Reverse Transcription (RT)

cDNA was synthesized in 50 μL volume containing 2 μg RNA, 10 mM Tris-HCl (pH 8.3), 75 mM KCl, 2.5 mM MgCl_2_, 1 mM dNTP (Promega), 10 mM DiThioThreitol (DTT), 1 mM antisense primer IRES-R (5′- TTT gCT gTA TTC AAC TTA ACA ATg-3′), 1U RNasin (Amersham, UK), 50 U of Moloney Murine Leukemia Virus Reverse Transcriptase (MMLV-RT) (Promega). The reaction was carried out at 42 °C for 30 min followed by a denaturation step of 5 min at 99 °C.

### 4.6. Amplification of CVB3 Wild-type and Sabin3-Like 5′UTRs

5′UTRs of the two CVB3 studied strains were amplified using primers 5′UTR-F (5′- TAT gAA TTC***TAA TAC gAC TCA CTA Tag*** gTA ACT TAg AAg TAA CAC A -3′) containing an EcoRI restriction site and the sequence of the T7 promotor; and 5′UTR-R (5′- TAT ggA TCC TTg CTg TAT TCA ACT TAA CAA TgA ATT gTA ATg TTT TAA -3′) including a BamHI restriction site. The amplification reaction was performed in a volume of 50 μL containing 100 ng cDNA, 25 mM Tris-HCl (pH 8), 50 mM KCl, 4 mM MgCl_2_, 200 μM dNTP, 40 ρmoles from each primer (5′UTR-F/5′UTR-R) and 1U Taq DNA polymerase (Promega). Distilled water was included as a negative control. Amplification was initiated by a denaturation step for 2 min at 95 °C followed by 5 cycles of 30 s denaturation at 95 °C, 30 s annealing at 48 °C and 30 s extension at 72 °C. Then, 25 cycles of 30 s at 94 °C, 30 s at 58 °C and 30 s at 72 °C were performed. Steps were followed by a final extension (10 min at 72°C). PCR products were electrophoresed in 1.5% agarose gels (Sigma) in 1X TBE buffer (containing 90 mM Tris HCl, 90 mM Boric Acid, 2 mM EDTA, pH 8.0) in the presence of 0.5 μg/mL of Ethidium Bromide (Sigma); and visualized by using Gel Doc 2000 system (Bio-Rad). Image processing and analysis of DNA bands were performed by using Quantity One software program (Bio-Rad).

### 4.7. Synthesis of the CVB3 3′UTR by Primers Hybridization and Extension

#### 4.7.1. Primers Design

Based on the sequence of the CVB3 complete genome published in the *GenBank* database under accession number *M88483* and referring to the 3′UTR secondary structure model previously proposed [48], primers 3′UTR-F (5′- TAT **gAA TTC*TAA TAC gAC TCA CTA TAg*** TTT TAg ATT AgA GAC AAT TTg AAA TAA TTT AgA TTg gCT TAA CCC TAC TgT gCT AAC CgA ACC AgA TAA Cgg -3′) and 3′UTR-R (5′- TAT **AAg CTTTTT TTT TTT TT**C CgC ACC gAA TgC ggA gAA TTT ACC CCT ACT gTA CCg TTA TCT ggT TCg gTT AgC ACA gTA ggg TTA AgC - 3′) were designed for synthesis of the 3′NCR by primers- hybridization and extension method. EcoRI restriction site/T7 promoter upstream and HindIII restriction site/poly (A) tail downstream were introduced into primer’s sequences.

#### 4.7.2. Synthesis of the CVB3 3′UTR

Primers- hybridization and extension technique was used to synthesize small DNA strands (100 to 250 nucleotides), that was the case of the 3′UTR (150 nucleotides). This technique consists briefly in reacting a pair of primers amplifying the gene of interest and designed so that an overlapping sequence of about 20 nucleotides between forward and reverse primers is incorporated. In practical terms, the reaction mixture was prepared as follows: Taq Buffer (1X) (Roche), 0.05 mM dNTPs, 100 pmol from each primer 3′NCR-F/3′NCR-R, 1U Taq polymerase (Roche) and the final volume (50 μL) was adjusted by adding sterile distilled water. Tubes were then incubated in a thermocycler (BioRad) according to the following thermal profile: 4 min at 72 °C, 6 min at 44 °C and 60 min at 70 °C. Amplification products were revealed by electrophoretic migration on an agarose gel containing Ethidium Bromide.

### 4.8. Cloning of the 5′ and 3′UTRs into the pUC19 Vector

Amplicons encoding 5′UTRS of both CVB3 studied strains were digested with EcoRI and BamHI (Roche Applied Science) (restriction sites were added by PCR). Similarly, the 3′UTR DNA was digested using EcoRI and HindIII (Roche Applied Science). Digestion products were, then, purified using the “QIA quick PCR Purification Kit” (Qiagen) and inserted into the pUC19 plasmid (Invitrogen) digested with the appropriate enzymes and purified using the “Wizard SV Gel and PCR Clean-Up system kit” (Promega). Ligation products were transformed in chemocompetent *E. coli* DH5α cells. Transformants were selected in LB agar supplemented with Ampicillin (100 μg/mL), X-Gal (64 μg/mL) and IPTG (0.2 mM). Blue-white selection was used to identify white clones containing inserts, while blue clones contained undigested cloning vector. White colonies were individually cultured in LB broth.

### 4.9. Screening of Transformed Clones

To analyze the cloned sequences, randomly chosen clones were tested by PCR-colony. A single colony isolated and added into 5 μL sterile water was used as template DNA in PCR reaction containing 15 μL premix solution. The amplification program was a pre-denaturation at 94 °C for 4 min followed by 30 cycles of denaturating at 94 °C for 1 min, annealing at 50 °C for 1 min, extension at 72 °C for 1 min, and a final extension at 72 °C for 10 min. After electrophoresis, an agarose gel was stained and DNA bands were compared to the expected sizes to determine positive clones. Additionally, these clones were sequenced using an ABI Prism BigDye Terminators Sequencing Kit (Applied Biosystems). Vector primers M13-F (5′- TgT AAA ACg ACg gCC AgT -3′) and M13-R (5′- CAg gAA ACA gCT ATg ACC- 3′) (New England Biolabs) were used for sequencing.

### 4.10. RNA and *In Vitro* Transcription

DNA sequences corresponding to the 5′UTRs cloned into pUC19 were amplified by PCR using T7 (5′- TAA TAC gAC TCA CTA TAg- 3′) as forward primer and 5′UTR-AUG (5′- *TgA TAC TTg AgC TCC***CAT** TTT gCT gTA TTC AAC TTA ACA ATg- 3′) as reverse primer. 15 nucleotides from the coding region and the initiating codon AUG were incorporated in the sequence of this reverse oligonucleotide. For this purpose, pUC19 recombinant plasmids (pUC19/5′UTR wild-type and pUC19/5′UTR *Sabin3-like*) were linearized using BamHI restriction enzyme and a PCR amplification reaction was performed using T7/5′UTR-AUG primers. The reaction mixture components were: 50 ng linearized pUC19/5′UTR, 1 μM from each primer, 0.2 mM dNTPs, Taq Buffer (1X), 5U Taq polymerase and the final volume of reaction was adjusted by adding distilled sterile water. PCR was carried out following a thermal profile consisting in a pre-denaturation step 3 min at 94 °C; 35 cycles of denaturating 1 min at 94 °C, annealing 1 min at 50 °C, extension 1 min at 72 °C, and a final extension step 10 min at 72 °C.

Both 5′UTRs and 3′UTR RNAs were then produced by *in vitro* transcription of amplicons containing T7 promoter sequence. The transcription reaction was conducted in a final volume of 100 μL using T7 RNA polymerase (New England Biolabs), 5 mM DTT, 4 mM NTP’s, Transcription Buffer (1X) (40 mM Tris–HCl pH 8.0; 25 mM MgCl_2_; 1 mM Spermidine) and 0.04U RNase Inhibitor (Applied Biosystems/Ambion) and incubated 2 h at 37 °C. During this incubation, and when a precipitate of RNA appears, 1U of Pyrophosphatase Alkaline (New England Biolabs) is added. Upon synthesis, RNAs were treated with RQ1 RNase-free DNAse (Promega) for 30 min at 37 °C.

RNAs were, then, precipitated with 2.5 M Lithium chloride (LiCl), incubated for 30 min at −20 °C, centrifuged at 13,000 rpm, 30 min at 4 °C, washed with 75% ethanol, resuspended in 50 μL of nuclease-free water and purified by size exclusion chromatography. RNA concentration was determined spectrophotometrically (NanoDrop Technology) and RNA integrity was monitored by electrophoresis on an agarose gel.

### 4.11. Periodate Oxidation and Fluorophore Coupling

Sodium periodate was used to oxidize the 3′ terminus of the RNA. The excess of the oxidant was removed by adding potassium chloride, and then the fluorescent dye, HiLyte Fluor™ 680, was added to label the 3′ terminus of RNA. The detailed procedure was as follows: the 5′UTR RNA was oxidised using sodium periodate (0.1 M) with sodium acetate (pH 5; 0.1 M) and incubated at 25 °C for 90 min. To stop the reaction, potassium chloride (250 mM) was added. The mix was incubated on ice for 10 min, then, centrifuged at 13000 rpm for 5 min at +4 °C. The supernatant was purified using Sephadex G50 column (GE Healthcare) to remove the excess of oxidant. Coupling was performed by addition of 8.6 mM HiLyte Fluor™ 680 (Eurogentec) in the presence of sodium acetate (pH 5; 0.1 M) then incubation at 25 °C for 4–17 hours in the dark. The molar ratio of the fluorescent dye to the RNA was 30:1. Fluorescent labeled RNA was precipitated by adding LiCl (7.5 M) and 2.5 vol ethanol, standing at −20 °C for 30 min, and then centrifuged (13,000 rpm at 4 °C) for 20 min. The precipitate was washed with 75% ethanol. Labeled RNA was resuspended in nuclease free water and the excess of fluorophore was removed by G50 Sephadex column purification (Roche Applied Science).

### 4.12. RNA-RNA Gel Shift Assay

An electrophoretic mobility shift assay or mobility shift electrophoresis, also referred as a gel shift assay, gel mobility shift assay or gel retardation assay, is a common affinity electrophoresis technique used to study protein-DNA, protein-RNA or RNA-RNA interactions. The principle behind EMSA relies on the fact that DNA-/RNA- protein complexes migrate slower than DNA or RNA alone in a native polyacrylamide or agarose gel. The speed at which different molecules move through the gel is determined by their size and charge, and to a lesser extent, their shape. This difference in electrophoretic separation of RNA-protein or RNA-RNA complexes can be visualized as a shift in migration of DNA or RNA band. For visualization purposes, the nucleic acid fragment is usually labeled with a radioactive or a fluorescent label since standard ethidium bromide staining is less sensitive than these methods and can lack the sensitivity to detect the nucleic acid if small amounts are used in these experiments.

In order to assess a possible interaction between CVB3 5′ and 3′UTRs, labeled 5′UTR wild-type and mutant RNAs and cold 3′UTR RNA were heat denatured in water for 2 min at 80 °C and slowly cooled to room temperature in Recon Buffer (2X) (60 mM Hepes pH 7.4, 0.2 M potassium acetate pH 7.6, 6 mM magnesium acetate, 4 mM DTT) for 10 min and then incubated 10 min at 4 °C. Constant amount of the labeled RNA (10 nM) was mixed with increasing concentrations of serially diluted (500, 250, 125, 62.5, 31.3, 15.6 nM) unlabeled 3′UTR RNA and incubated 15 min at 4 °C and, in parallel, the same assay was carried out with an incubation of samples during 30 min at 37 °C. Samples were then mixed with loading buffer (10% sucrose or 30% glycerol) and separated in a native 4% polyacrylamide gel (1 mL THEM buffer (10X) (containing 340 mM Tris, 570 mM Hepes, 1 mM EDTA, 25 mM MgCl_2_), 1 mL acrylamide 40%, 100 μL APS 10%, 10 μL TEMED and 8 mL H_2_O). THEM (1X) was used as a running buffer and the gel was subsequently visualized and analyzed by Odyssey scanner (Li-COR Odyssey, Biosciences). In parallel to CVB3 RNAs, the 5′ and 3′ non coding regions of HCV were used as a positive control for the shift reaction as previously described by Romero-Lòpez and Berzal-Herranz [50].

## 5. Conclusion

In summary, 5′–3′ ends interactions could take place through direct RNA-RNA contacts, through protein bridges mediating RNA circularization or both. The present results support the existence of a protein-dependent 5′–3′ communication in the CVB3 genome that involves RNA elements essential for viral translation. It is tempting to speculate that one or more of these host proteins may be involved in interacting simultaneously with the 5′ and 3′UTRs, thus bringing about circularization of the mRNA. It is also possible that some of this ITAF interaction, together with long range RNA-RNA interaction, might contribute to the efficient IRES activity of CVB3 RNA. Therefore, a further investigation by functional analysis to confirm the identity and the role of these proteins in mediating cross-talk between the 5′ and 3′ ends of CVB3 RNA may provide new insights into our understanding of the role of protein-RNA and RNA-RNA interactions in the regulation of viral translation and its relationship to cardiovirulence.
